# Identification and characterization of linear epitopes of monoclonal antibodies against the capsid proteins of small ruminant lentiviruses

**DOI:** 10.3389/fmicb.2024.1452063

**Published:** 2024-08-01

**Authors:** Xiaohua Ma, Min Gao, Xiangmin Zhang, Weiwei Ma, Fei Xue, Xue-Feng Wang, Xiaojun Wang

**Affiliations:** ^1^State Key Laboratory for Animal Disease Control and Prevention, Harbin Veterinary Research Institute of Chinese Academy of Agricultural Sciences, Harbin, China; ^2^Institute of Western Agriculture, The Chinese Academy of Agricultural Sciences, Changji, China

**Keywords:** small ruminant lentiviruses (SRLVs), maedi-visna virus (MVV), caprine arthritis encephalitis virus (CAEV), monoclonal antibodies (mAbs), capsid protein

## Abstract

Maedi-visna virus (MVV) and caprine arthritis encephalitis virus (CAEV) are members of a group of genetically highly homologous lentiviruses collectively referred to as small ruminant lentiviruses (SRLVs). SRLVs can infect sheep, goats and other small ruminants, causing multisystemic disease with progressive and persistent inflammatory changes, severely reducing animal productivity and impeding animal trade. The capsid protein of SRLVs, p28, is highly conserved among strains and is a commonly used marker for the detection of SRLVs. In this study, two monoclonal antibodies (mAbs), designated G8F7 and A10C12, against p28 were generated using a recombinant p28 protein expressed in *Escherichia coli* as an immunogen. Functional analysis showed that these two monoclonal antibodies could be used in iELISA, immunofluorescence assays (IFA) and western blot assays to detect p28 or Gag precursor proteins of SRLVs. Two linear epitopes, ^61^GNRAQKELIQGKLNEEA^77^ (E61-77) and ^187^CQKQMDRVLGTRVQQATVEEKMQACR^212^ (E187-212), which are recognized by G8F7 and A10C12, respectively, were identified through truncation of the GST-fused p28. Amino acid sequence alignment showed that the epitope E61-77 is conserved among SRLVs, with a dominant mutation site (K72R) that does not disrupt recognition by G8F7. E187-212 was found to exhibit variability among SRLVs, but the majority of mutant epitopes are recognized by A10C12, with the exception of a mutant epitope from an isolate with undefined subtypes from *Ovis aries*, which was not recognized. These findings may facilitate future study of SRLVs and promote the development of methods for the detection of these viruses.

## 1 Introduction

Small ruminant lentiviruses (SRLVs), which belong to the lentivirus genus of the Retroviridae family, include maedi-visna virus (MVV) and caprine arthritis encephalitis virus (CAEV). Other members of the lentivirus genus include the human immunodeficiency virus-1 (HIV-1), human immunodeficiency virus-2 (HIV-2), simian immunodeficiency virus (SIV), feline immunodeficiency virus (FIV), bovine immunodeficiency virus (BIV), equine infectious anemia virus (EIAV), Puma lentivirus (PLV) and Jembrana disease virus (JDV). MVV was the first lentivirus to be discovered, and was originally isolated from Iceland. Early studies observed that the virus mainly infects sheep, causing slowly progressive interstitial pneumonia (the corresponding clinical signs are called Maedi in Iceland) and central nervous system inflammation (the clinical signs are called Visna in Iceland), as well as occasional joint and/or mastitis, hence the name Maedi-Visna (MV) for the disease (Kennedy et al., 1968; Minguijon et al., 2015). MV is also known as ovine progressive pneumonia (OPP) in the United States. CAEV has a similar genome structure to MVV (Saltarelli et al., 1990), and initial studies concluded that it infected only goats, causing arthritis and encephalitis, so the resulting disease was named caprine arthritis/encephalitis (CAE) (Cork et al., 1974; Crawford et al., 1980). Subsequently, an increasing number of molecular epidemiologic studies and clinical case analyses have shown that the two viruses are phylogenetically close and can be transmitted between species in goats and sheep (Banks et al., 1983; Karr et al., 1996; Shah et al., 2004). This suggests that MVV and CAEV may represent quasispecies of SRLVs with different host adaptations and pathogenicity, rather than different virus species or etiologic agents (Pasick, 1998). Over the past two decades, these two viruses have often been referred to collectively as SRLVs, and the associated diseases have been referred to as SRLVs infection (Minguijon et al., 2015). However, currently, MV and CAE are still considered to be and notified as two separate diseases by the World Organisation for Animal Health (WOAH) and the Chinese Ministry of Agriculture, and both diseases are included in the list of notifiable terrestrial and aquatic animal diseases by WOAH. The diseases are endemic in many countries around the world, including Japan (Oguma et al., 2014), Canada (Santry et al., 2013), Spain (Gayo et al., 2018), Poland (Olech et al., 2022), India (Kumar et al., 2022), Jordan (Hailat et al., 2022), Italy (Arcangeli et al., 2022) and China (Zhao et al., 2021). Although the majority of SRLVs-infected animals have no typical clinical symptoms (arthritis, mastitis, pneumonia, or encephalomyelitis), these animals remain persistently infected lifelong, and SRLVs infections cause significant economic losses due to weight loss, low birth weight, decreased production of milk and trade restrictions (Juste et al., 2020). As a result, many countries have established MV and CAE specific control and eradication strategies (Tavella et al., 2018; Nardelli et al., 2020).

Similar to other lentiviruses, SRLVs also possesses three structural genes, *gag*, *pol* and *env*. The *gag* gene encodes a Gag precursor protein (p55) that is cleaved by viral proteases into the matrix protein (MA, p17), capsid protein (CA, p28; also known as p25 or p27 in some literature), and nucleocapsid protein (NC, p14). The *pol* gene encodes the viral enzymes, including protease (RP), dUTPase (DU), reverse transcriptase (RT), and integrase (IN). The *env* gene encodes the envelope glycoproteins (Env), including surface glycoproteins (SU, gp135) and transmembrane protein (TM, gp38). In addition, three accessory genes (*vif*, *rev*, and *vpr-like*) have been identified in SRLVs, and encode proteins with similar functions to the corresponding accessory proteins of other lentiviruses. SRLVs have wide genetic diversity, with the *gag* and *pol* genes being relatively conserved, while the env gene show a high degree of variability (Olech et al., 2018; Bazzucchi et al., 2021; Arcangeli et al., 2022). Based on a genetic analysis of the *gag* and *pol* sequences, SRLVs was reclassified into five genotypes, A–E (Shah et al., 2004), and further divided into more than 20 subtypes (Bazzucchi et al., 2021; Olech et al., 2022). Commonly, the structural proteins Gag and Env of lentiviruses are the main targets of the humoral immune response and are frequently used as antigens for serological diagnosis. Notably, CA is the most abundant protein in the lentiviral virion and is highly conserved within each lentivirus. The amount of CA protein associated with viral particles is commonly used as an indicator of lentiviral load, and the CA is therefore recognized as a virological biomarker of lentiviral infection and replication (Gray et al., 2018). In SRLVs, p28, gp135 and gp38 are the main immunogenic proteins, and p28 in particular is widely used as a viral antigen in serological diagnostics (De Andres et al., 2005; Kalogianni et al., 2021).

In this study, we expressed and purified recombinant SRLVs p28 protein, then generated two mAbs against SRLVs p28. Two linear p28 B-cell epitopes, ^61^GNRAQKELIQGKLNEEA^77^, and ^187^CQKQMDRVLGTRVQQATVEEKMQACR^212^, were subsequently identified with western blot (WB) analysis using these mAbs. We also analyzed the conservation of these epitopes among epidemic SRLVs. These results provide valuable information for the research into and diagnosis of SRLVs.

## 2 Materials and methods

### 2.1 Cells, virus, mice and vector

Human embryonic kidney 293 T (HEK293T) and Hela cells were cultured in Dulbecco’s modified Eagle’s medium (DMEM) (ThermoFisher Scientific, United States) containing 10% heat-inactivated fetal bovine serum (FBS), at 37°C in a humidified incubator under 5% CO_2_. The SP2/0 myeloma cell line was maintained in RPMI-1640 (Sigma-Aldrich, United States) supplemented with 20% heat-inactivated FBS (Ausbian, Australia). The SRLVs (MVV) strain SRLV-X was stored in the Harbin Veterinary Research Institute, Chinese Academy of Agricultural Sciences (after sequencing the virus, evolutionary analysis showed that this virus strain belonged to genotype A2, GenBank accession number: PP854830). Six-week-old specific pathogen-free (SPF) grade BALB/c female mice were provided by the Animal Experiment Center of Harbin Veterinary Research Institute. The eukaryotic expression vector pcDNA3.1-HA, the prokaryotic expression vector pET-30a(+) and pGEX-6P-1 were maintained in our laboratory. The plasmid pcDNA-Gag-HA, expressing a codon-optimized SRLV gag gene (derived from SRLV-X) was generated using gene synthesis and insertion into the pcDNA3.1-HA vector. Three SRLV Gag prokaryotic expression plasmids (pET30-Gag-SC; pET30-Gag-XJ; pET30-Gag-NM) were generated by synthesizing gag gene sequences from three Chinese SRLV strains isolated from Sichuan (GenBank accession number: KT214469.1, from goat), Xingjiang (GenBank accession number: MZ313871.1, from sheep), and Inner Mongolia (GenBank accession number: MW248464, from sheep), followed by subcloning into the pET-30a(+) vector.

### 2.2 Expression and purification of recombinant SRLVs p28

The full-length p28 coding sequence was amplified from SRLVs-X using reverse transcription (RT)-PCR. The purified PCR products were sequentially cloned into pGEX-6P-1 and pET-30a(+) vector, and generated the recombinant plasmids pGEX-6P-GST-p28 and pET-30a-His-p28. To obtain the GST-p28 and His-p28 fusion proteins, pGEX-6P-GST-p28 and pET-30a-His-p28 were transformed into *E. coli* Rosetta (DE3) competent cells for protein expression with the addition of 0.3 mM isopropyl-β-D-1-thiogalactopyranoside (IPTG), and were grown for 8 h at 30°C in Luria-Bertani (LB) medium containing ampicillin or kanamycin, respectively. Bacterial cells were then centrifuged at 5,000 × g for 10 min, washed with phosphate-buffered saline (PBS, pH 7.4), and resuspended in a final volume of 40 mL PBS (for GST-p28) or 40 mL binding buffer (pH 8.0, for His-p28), and lysed using sonication. Recombinant GST-p28 and His-p28 were purified using a GST-tag Protein Purification Kit (BeyoGold, China) or High Affinity Ni-Charged Resin (GenScript, China), respectively. The purified proteins were then analyzed with sodium dodecyl sulfate-polyacrylamide gel electrophoresis (SDS-PAGE). Finally, the proteins were stored at −80°C.

To map the p28 antigenic epitopes recognized by the prepared mAbs, a series of truncated p28 fragments were constructed based on pGEX-6P-GST-p28, and the recombinant protein was expressed in *E. coli* Rosetta (DE3) cells as described above. Bacterial cells transformed with recombinant plasmids containing truncated p28 fragments were then lysed, and the reactivity of the prepared mAbs against these fragments was determined with WB.

### 2.3 Preparation of mAbs

Six-week-old female BALB/c mice were injected subcutaneously with 100 μg of purified recombinant His-p28 protein three times, at 3 weeks intervals. Serum samples were collected and the antibody responses were analyzed with indirect ELISA (iELISA) coated with recombinant GST-p28 protein. The splenocytes of the mice were isolated and fused with SP2/0 myeloma cells under the action of PEG solution (Sigma-Aldrich, Germany), and the same dose of protein without adjuvant was injected subcutaneously 72 h prior to this step. The fused cells were then plated into 96-well plates and cultured in HAT (Hypoxantin, Aminopterin, Thymidin) selection medium (Sigma-Aldrich, United States). Seven days later, positive hybrids were identified with iELISA. After three-round subcloning, a single cell with a higher OD_450_ nm value was selected. This selected single cell was expanded and cultured, the cell culture fluid was identified with WB, and hybridomas stably producing mAbs were generated. The isotypes of the mAbs were determined using a commercial antibody subtype identification kit (Southern Biotech, China). For the production of mAbs, positive hybridomas were used to immunize Freund’s incomplete adjuvant-primed BALB/c mice to produce ascites, and the ascites were collected daily with a sterile syringe 1 week after injection of hybridoma cells. The mAbs in the ascites were purified using an antibody purification kit (GE Healthcare, United States) and WB, IFA and iELISA were used to analyze the specificity of the prepared mAbs.

### 2.4 iELISA

Purified GST-p28 protein was used to coat flat-bottom polystyrene plates (100 ng/well) with overnight incubation at 4°C. The plates were washed with PBST (0.05% Tween in PBS, v/v) three times before being blocked with 5% BSA in PBS for 2 h at 37°C. After another washing step, 100 μL of hybridoma supernatants or a gradient dose of purified monoclonal antibody were added into the plates, and samples were then incubated at 37°C for 2 h. After three washes, 1:4000 dilutions of horseradish peroxidase (HRP)-conjugated goat anti-mouse IgG (KPL, United Sates) were added, and samples were incubated for 45 min at 37°C. After a final wash, a chromogenic substrate solution (TMB) (Abcam, United Kingdom) was added and the samples were incubated for 10 min, after which 2 M H_2_SO_4_ was added to stop the reaction. The plates were then measured at 450 nm using an automated ELISA plate reader (Bio Tek, United Sates).

### 2.5 Western blot

To assess the reactivity of the prepared mAbs with recombinant p28 proteins or to identify the minimal epitopes required for the recognition of p28, prokaryotic expression samples of p28 proteins or truncated p28 fragments were subjected to SDS-PAGE electrophoresis and then transferred onto nitrocellulose filter membrane (Millipore, Germany). The membranes were then blocked with 5% skimmed milk for 2 h at room temperature and were incubated with anti-p28 mAbs (hybridoma supernatant) or anti-GST antibody overnight at 4°C. After three washes with TBST, the membranes were incubated with anti-mouse IgG-DyLight 800 (18,000 dilution) for 1 h. After a final washing step, the membranes were screened using the LI-COR Odyssey Imagining System (LI-COR, United States).

To assess the reactivity of the prepared mAbs with SRLVs Gag, HEK293T cells were transfected with pcDNA-Gag-HA or empty vector using PolyJet DNA transfection reagent (SignaGen, United States), following the manufacturer’s instructions. At 48 h post-transfection (hpt), cells were collected and lysed. The proteins were subjected to electrophoresis on a 12% SDS-PAGE Gel, and a WB was conducted as described above.

### 2.6 IFA

HeLa cells were transfected with pcDNA-Gag-HA plasmid or empty vector using PolyJet DNA transfection reagent (SignaGen, United States). At 36 hpt, the cells were then washed three times with PBS and fixed with 4% paraformaldehyde for 30 min and permeabilized with 0.1% Triton X-100 for 15 min. The cells were then washed three more times and incubated with blocking solution (5% skim milk) for 1 h. Samples were incubated with the prepared mAbs as primary antibodies for 2 h at room temperature, washed three times with PBS, and then incubated with Alexa Flour 488-conjugated secondary antibodies (Thermo Fisher Scientific, United States) for 1 h in the dark. The cell nuclei were stained with 4′, 6-diamidino-2-phenylindole (DAPI), and fluorescence signals were analyzed using a confocal microscope.

### 2.7 Conservation analysis of the identified epitopes

572 SRLVs p28 amino acid sequences were obtained from Genbank (Supplementary data 1). To assess the degree of conservation of the E61-77 and E187-212 epitopes, multiple alignments of the E61-77 and E187-212 amino acid sequences were performed using the Clustal W method within DNASTAR software version 7.0, and then the online software WebLogo^1^ was used to construct WebLogo diagrams.

## 3 Results

### 3.1 Expression and purification of the recombinant GST-p28 and His-p28 proteins

**Figure 1:**
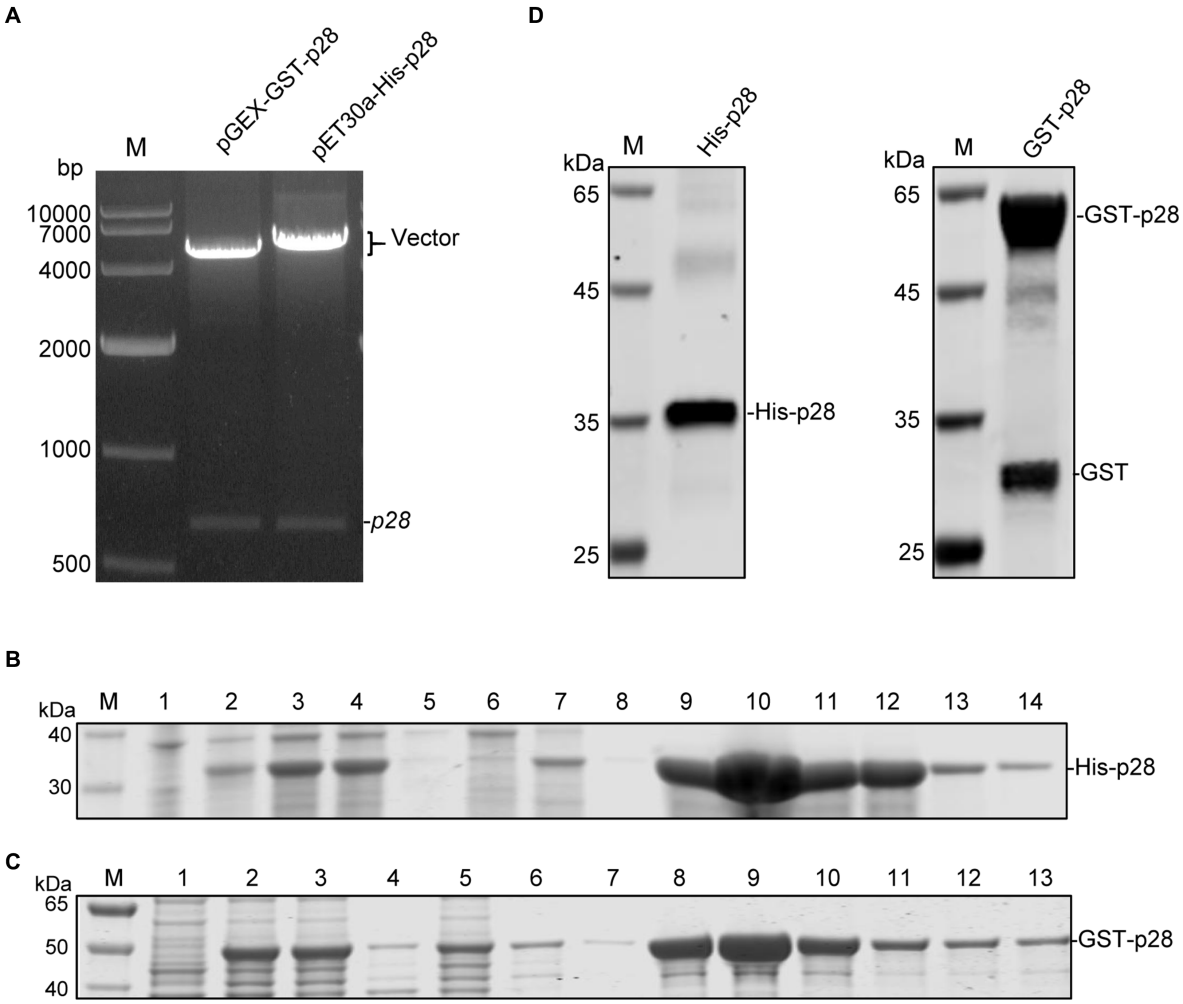
Expression and purification of recombinant SRLVs p28 containing a His-tag or a GST-tag. **(A)** Identification of recombinant expression vectors pGEX-GST-p28 and pET30a-His-p28 by *EcoR* I/*Xho* I digestion. Lane M: DNA molecular weight marker. **(B)** SDS-PAGE analysis of recombinant His-p28 protein expression in *E. coli* Rosetta. Lane M, protein marker; lane 1, lysates from *E. coli* Rosetta (DE3) transformed with the empty vector pET30a; lane 2, lysates from *E. coli* Rosetta (DE3) transformed with the pET30a-His-p28 without IPTG induction; lanes 3–5, bacterial lysate (lane 3), cell supernatant (lane 4) and cell precipitation (lane 5) after sonication of *E. coli* Rosetta (DE3) transformed with pET-30a-His-p28 with IPTG induction; lanes 6–14, purification of recombinant His-p28 protein using Ni2^+^-NTA resins; lane 6, flow-through fluid; lane 7–8, cleaning fluid; lane 9–14, purified His-p28. **(C)** SDS-PAGE analysis of GST-p28 expression in *E. coli* Rosetta. Lane M, protein marker; lane 1, lysates from *E. coli* Rosetta (DE3) transformed with the empty vector pGEX-6p-1; lanes 2–4, bacterial lysate (lane 2), cell supernatant (lane 3), and cell precipitation (lane 4) after sonication of *E. coli* Rosetta (DE3) transformed with pGEX-GST-p28 with IPTG induction; lanes 5–13, purification of recombinant GST-p28 protein using GST sepharose resin; lane 5, flow-through fluid; lane 6–7, cleaning fluid; lane 8–13, purified GST-p28. **(D)** Western blotting analysis of purified His-p28 and GST-p28 using anti-His or anti-GST antibodies.

To express SRLVs p28 protein, the p28 coding sequence of the SRLVs-X strain was first amplified using RT-PCR, and was then cloned into the *EcoR* I and *xho* I restriction sites of the pET-30a(+) and pGEX-6p-1 vectors, respectively. The recombinant positive plasmids pET-30a-His-p28 and pGEX-6P-GST-p28 were identified with restriction enzyme digestion (Figure 1A) and sequencing, and then were transformed into *E.coli* Rosetta (DE3) cells for fusion protein expression after IPTG induction. SDS-PAGE analysis confirmed the expression of the recombinant His-p28 and GST-p28 proteins with the predicted molecular mass, and these proteins were found to be present mainly in the cell lysate supernatant after sonication. The His-p28 and GST-p28 recombinant proteins were then purified using nickel column affinity chromatography and GST sepharose resin, respectively, and high purity recombinant His-p28 and GST-p28 were obtained (Figures 1B,C). WB analysis indicated that the recombinant His-p28 and GST-p28 proteins reacted specifically with the anti-His and the anti-GST antibodies, respectively (Figure 1D).

### 3.2 Preparation and characterization of mAbs against SRLVs p28

**Figure 2:**
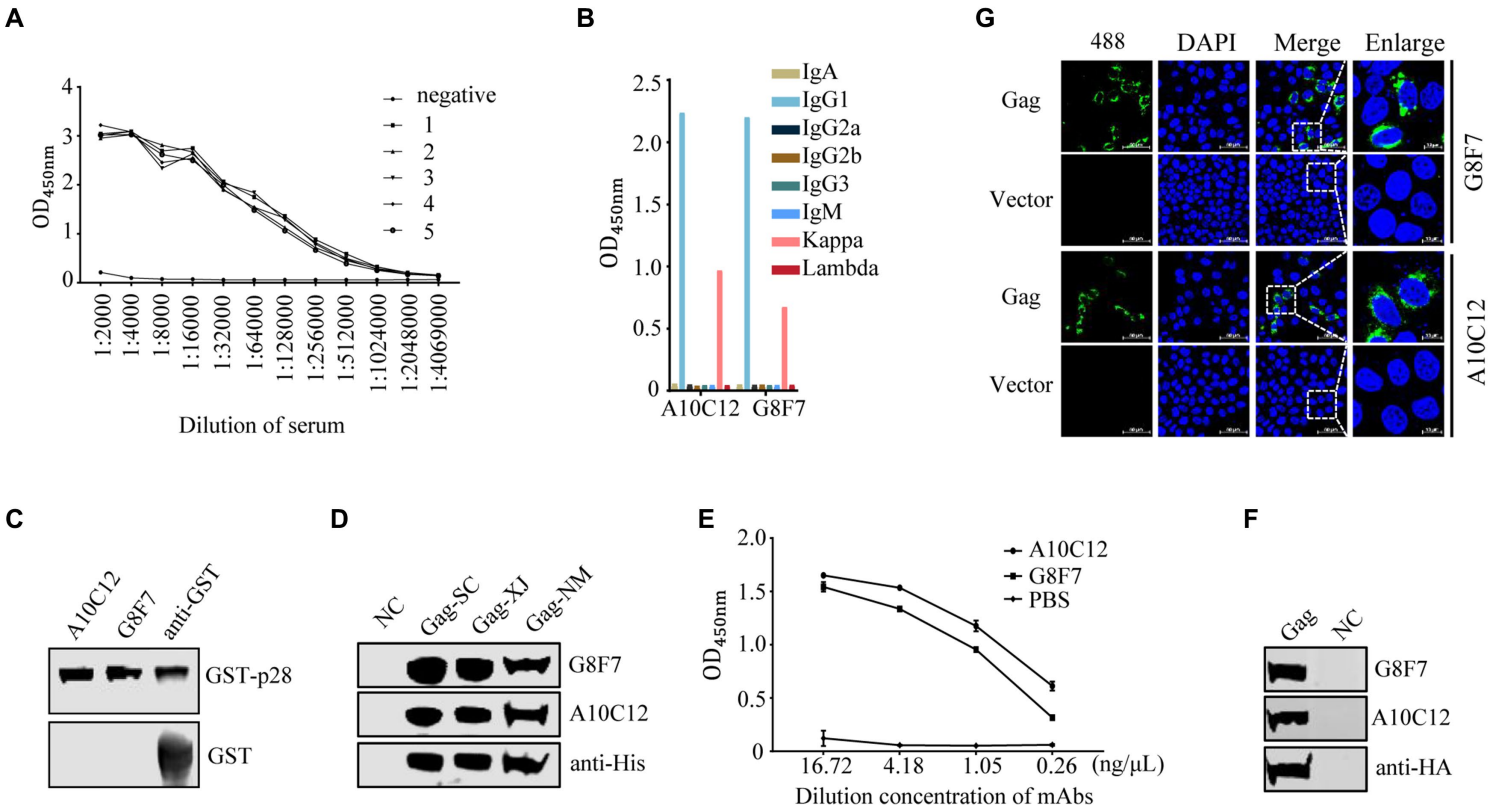
Screening and characterization of monoclonal antibodies. **(A)** Detection of serum antibody titers. One week following the third immunization, serum samples were collected from each of the five immunized mice. Serially diluted serum samples were tested with an indirect ELISA using purified GST-p28 protein as a coating antigen. 1–5 represent serum samples derived from the five mice. Serum samples from an unimmunized mouse were as used a negative control (NC). **(B)** Monoclonal antibody isotype identification. The isotypes of prepared mAbs were determined using a commercial antibody subtype identification kit. **(C)** Reactivity of the prepared mAbs with prokaryotic recombinant GST-p28 protein. A WB assay was performed to detect purified GST-p28 from *E. coli* using the indicated antibodies as primary antibodies (upper). GST protein was used as an NC (lower). **(D)** Reactivity of the prepared mAbs with prokaryotic SRLVs Gag protein. A WB assay was performed to detect Gag expression in *E. coli* Rosetta (DE3) cells transformed separately with the pET30a-Gag-SC, pET30a-Gag-XJ, or pET30a-Gag-NM, using the indicated antibodies as primary antibodies. **(E)** An iELISA was performed using purified GST-p28 protein as the coating antigen (200 ng/well) to detect the serially diluted mAb G8F7 or A10C12 (PBS was used as a NC). Each sample was tested in triplicate. **(F-G)** Reactivity of the prepared mAbs with eukaryotic SRLVs Gag protein. **(F)** HEK293T cells were transfected with pcDNA-Gag-HA or empty vector pcDNA-HA (as a NC). The cells were collected at 48 hpt and were lysed for the detection of Gag using the indicated antibodies as primary antibodies. **(G)** Hela cells were transfected with pcDNA-Gag-HA or empty vector pcDNA-HA (as a NC). At 36 hpt, cells were fixed and stained with the indicated antibodies as primary antibody and Alexa Fluor 488-conjugated anti-mouse secondary antibody (green), and nuclei were stained with DAPI (blue). Protein expression was analyzed by confocal microscopy.

The purified recombinant His-p28 proteins were used as immunogen to immunize five female BALB/c mice. After three immunizations, the anti-serum titers of mice to which His-p28 proteins had been administered were determined with iELISA using purified GST-p28 proteins as an antigen. The results showed that all immunized mice had high antibody titers to the immunogens compared to the pre-immunization serum (Figure 2A). Two hybridoma cell lines secreting antibodies against SRLVs p28 were obtained with iELISA and three-round subcloning with limiting dilution, and the anti-p28 antibodies secreted by these cell lines were further biochemically characterized. Antibody isotype assays determined that two mAbs (G8F7 and A10C12) belonged to the IgG1κ type (Figure 2B). These two hybridoma cell lines were then injected into the peritoneal cavity of mice for the production of mAbs in the ascites. The results of the subsequent WB assay showed that both mAbs reacted with the recombinant GST-p28 protein (Figure 2C) and the prokaryotically expressed SRLVs Gag proteins from three Chinese isolates (Figure 2D). In addition, these mAbs could be used in an iELISA to detect p28 (Figure 2E). Furthermore, WB (Figure 2F) and IFA (Figure 2G) analyses demonstrated that these mAbs also recognized eukaryotically expressed SRLVs Gag.

### 3.3 Identification of the epitopes recognized by the two mAbs

**Figure 3:**
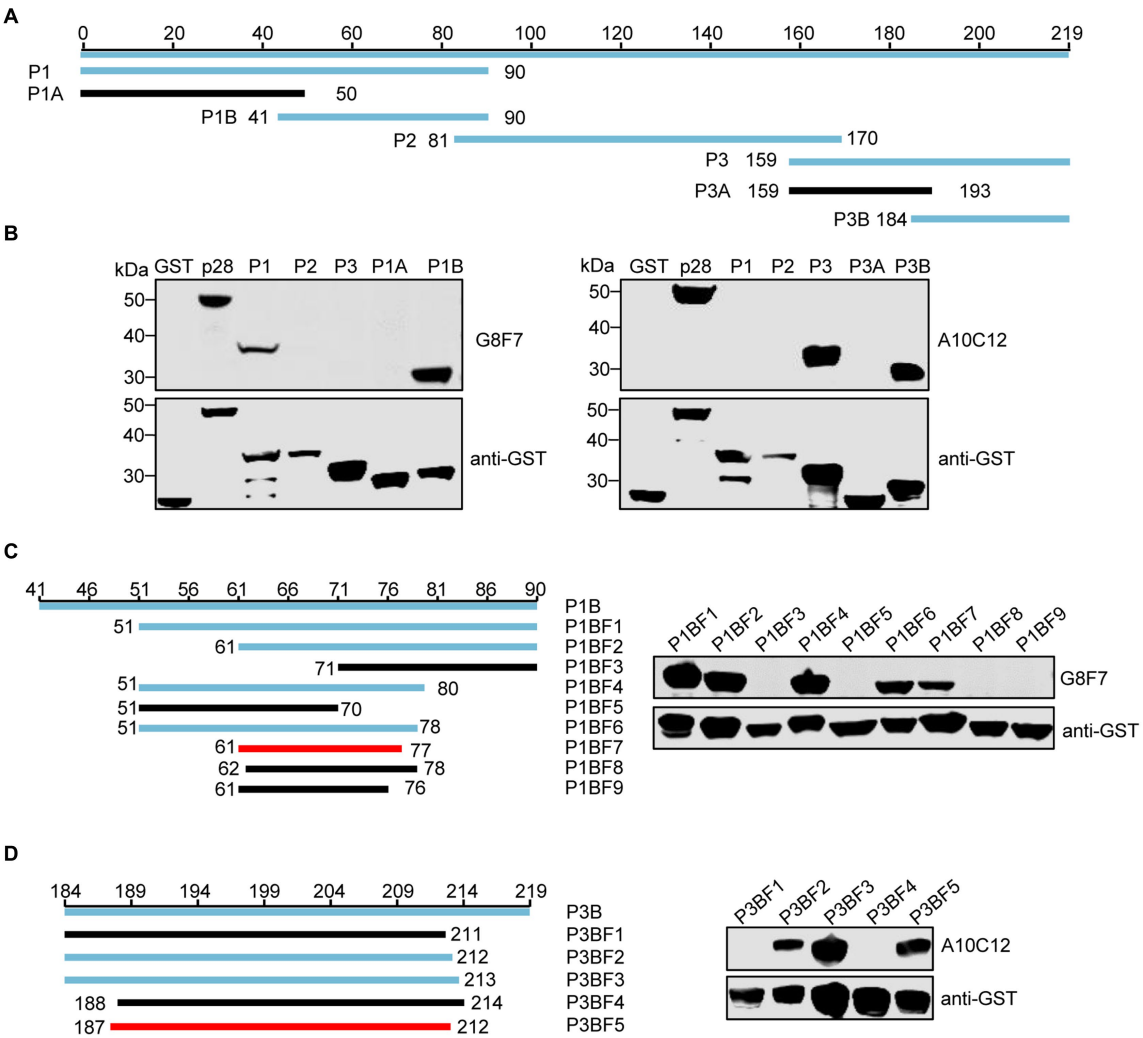
Western blot epitope mapping. **(A)** Schematic representation of the p28 fragments used for epitope mapping. The blue lines represent p28 fragments that react with mAb G8F7 or A10C12 and the black lines represent p28 fragments that do not react with either mAb. The numbers represent the positions of the amino acids in p28. **(B)** As shown in panel A, GST-p28 was segmentally expressed in *E. coli* Rosetta (DE3) and then subjected to WB using mAbs G8F7 and A10C12 as primary antibodies. **(C)** Identification of the minimal epitope recognized by G8F7. The left panel shows the truncated p28 fragment based on the P1B fragment. The numbers represent the positions of the amino acids in the p28. The blue and black lines represent fragments reacting with or without mAb G8F7, respectively. The red line represents the minimal fragment recognized by G8F7. The right panel shows the reactivity of GST-tagged p28 truncated fragments (as shown in the left panel) with the mAb G8F7 analyzed using WB. The anti-GST antibody was used to determine the loading dose of each sample. **(D)** Identification of the minimal epitope recognized by A10C12 as described in **C**.

To identify the epitopes recognized by the G8F7 and A10C12 mAbs, we used a truncated mutagenesis method to express SRLVs p28 in three fragments in the GST-p28 expression vector. The fragments were named P1 (1–90 aa), P2 (81–170 aa), and P3 (159–219 aa) (Figure 3A). WB assays demonstrated that G8F7 recognized the P1 fragment and A10C12 recognized the P3 fragment (Figure 3B). To further characterize the epitopes recognized by these two mAbs, we performed several rounds of truncating mutagenesis based on P1 and P3 (Figures 3C,D), and finally confirmed that the minimal linear epitopes recognized by these mAbs were ^61^GNRAQKELIQGKLNEEA^77^ (recognized by G8F7, named E61-77) and ^187^CQKQMDRVLGTRVQQATVEEKMQACR^212^ (recognized by A10C12, named E187-212).

### 3.4 Conservation of the identified epitopes in SRLVs

**Figure 4:**
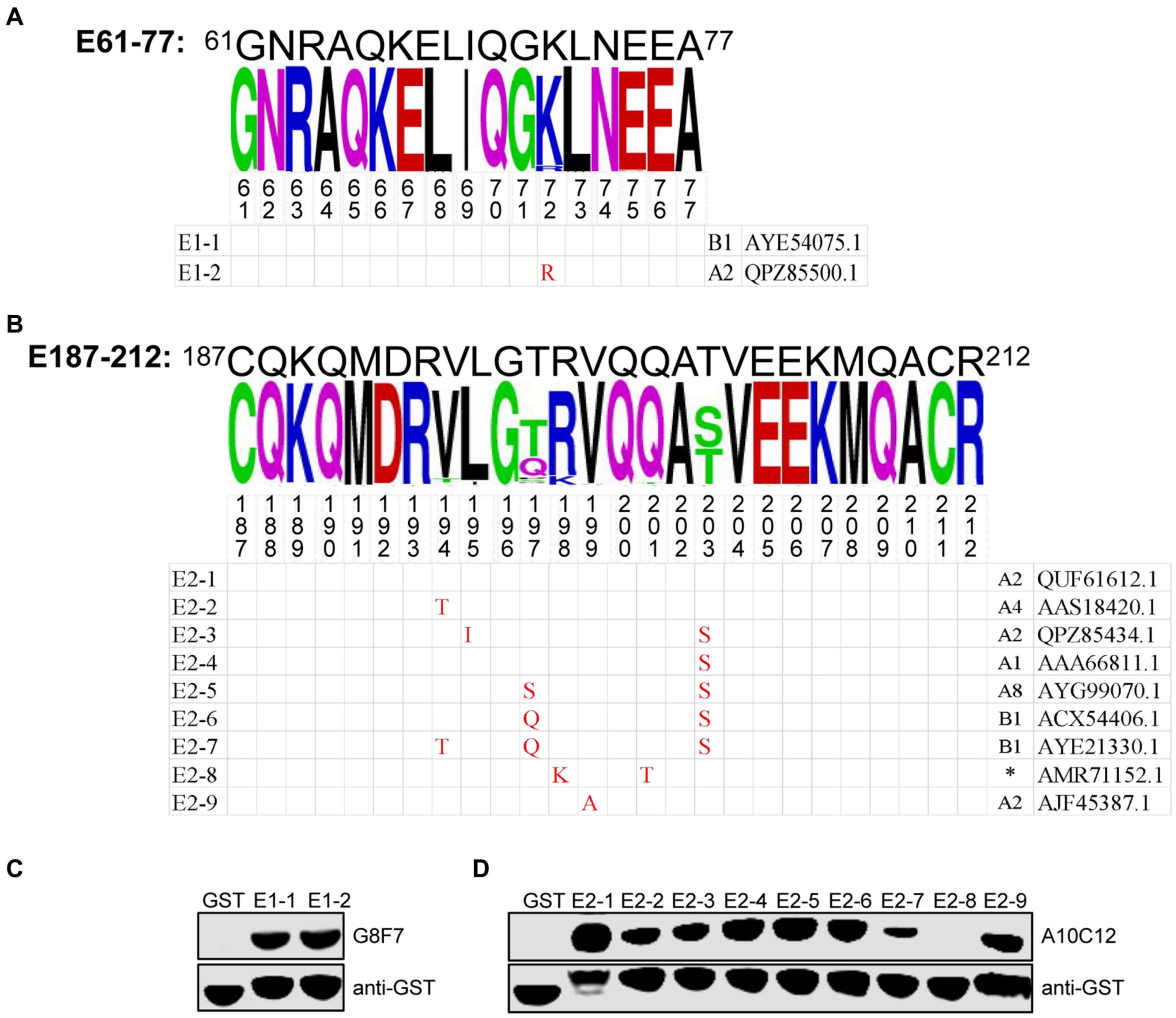
Conservation analysis of epitopes E61-77 and E187-212. **(A,B)** Upper panel, WebLogo presentation of the variability of epitopes E61-77 (A) and E187-212 **(B)** based on the alignment of 572 amino acid sequences of SRLVs p28 downloaded from the NCBI database. The height of each stack corresponds to the level of amino acid conservation at that position. If the amino acid is invariant, only one letter is shown; if the amino acid is variable, the most common substitutions are noted. The figures show the mutation hotspots in the E61-77 and E187-212. Lower panel, natural epitope sequences containing the mutation hotspots indicated in the upper panel were constructed as GST fusion proteins. The combination of letters and numbers in the left column in each sequence indicates the name of the epitope, and the Genbank accession number of the p28 amino acid sequence corresponding to each sequence and the SRLVs subtype to which it belongs are shown in the right column. Red font indicates residues that are mutated compared to the E61-77 or E187-212 sequence. Amino acid residues that are consistent with the sequence of E61-77 or E187-212 are shown as a blank cell. **(C,D)** GST fusion proteins containing the E61-77 **(C)** or E187-212 **(D)** epitope sequences with the mutation hotspots indicated in the **(A,B)** were expressed in *E. coli* Rosetta (DE3) and their reactivity with the mAbs G8F7 and A10C12 was analyzed with WB. GST proteins were used as specific controls. The GST antibody was used to determine the loading dose of each sample. (*) indicates that the SRLVs subtype is not defined for that strain.

We next assessed the conservation of E61-77 and E187-212 among SRLVs isolates. To this end, 572 SRLVs p28 amino acid sequences containing these two epitopes were downloaded from Genbank and aligned. We found that E61-77 is highly conserved among different isolates of SRLVs, with only one mutational hotspot (K72R), while there are seven mutational hotspots (V194T, L195I, T197Q(S), R198K, V199A, Q201T, T203S) present in E187-212 (Figures 4A,B). Subsequently, GST fusion proteins were prepared, containing E61-77 and E187-212, with these mutation sites naturally occurring in various SRLVs isolates. WB analysis showed that the K72R substitution within the E61-77 epitope did not affect its reactivity with G8F7, and that the E187-212 epitope with combined R198K and Q201T substitutions (representing an SRLVs strain with undefined subtypes isolated from *Ovis aries* in Jordan) lost reactivity with A10C12. No significant change in the reactivity of the other E187-212 variants with A10C12 was observed (Figures 4C,D).

## 4 Discussion

Lentiviruses, including SRLVs, integrate into the host genome upon infection. The host is unable to eliminate the virus and a lifelong infection is established. SRLVs infections are widespread and common, there are no specific antiviral treatments for these diseases, and the development of an effective and safe vaccine remains a formidable hurdle in lentivirus research. In the absence of a vaccine, early diagnosis and screening are important strategies for the prevention and control of lentiviral infections in farm animals. The CA protein is the most abundant viral protein in the lentivirus virion and is a well-established viral marker for diagnosis. Here, we generated two monoclonal antibodies against the SRLVs CA protein, p28, using the recombinant p28 protein expressed in *E. coli* as immunogen, and characterized the epitope sequences recognized by these two antibodies.

The CA is highly conserved within each lentivirus and is also the most immunogenic protein in lentiviruses. Antibodies to CA are usually the first detectable in the lentivirus-infected host and can be present for long periods of time. Therefore, this protein and antibodies against it are considered excellent candidates for the detection of lentiviral infection, especially when new mutant strains emerge and when specificity tests are not available. In this study, we used an expression system in *E. coli* to generate recombinant p28, of the A2 genotype of SRLVs, which is the main genotype currently prevalent in China (Zhao et al., 2021; Wu et al., 2022). Recombinant CA proteins are widely used for serology and antigen detection of retroviral infection, including as ELISAs (Bai et al., 2019), agar gel immunodiffusion (AGID) tests (Alvarez et al., 2007) and gold immunochromatographic test strips (Zhang et al., 2024). Therefore, it is possible that the recombinant p28 prepared in this study may be effective as a detection antigen in serological diagnostic methods for SRLVs infection.

mAbs against the CA protein are extremely important tools for both basic research and diagnosis of lentiviral infections. However, there is very little literature on mAbs against the SRLVs p28 (Brandao et al., 2013). Previously, Brandao et al. (2013) generated mAbs against CAEV p28 using the whole virus as the immunogen. They demonstrated that these antibodies recognize the p28 and Gag precursor proteins as well as the intermediate cleavage products from CAEV-infected cells, but the epitope sequences recognized by these mAbs were not identified (Brandao et al., 2013).

In this study, we generated two mAbs against p28, G8F7 and A10C12, using recombinant p28 as the immunogen. To ensure the specificity of the screening mAbs, we prepared two recombinant p28 proteins, His-p28 and GST-p28, the former as an immunogen to immunize mice and the latter as a detection antigen to screen p28-specific mAbs, effectively eliminating the occurrence of false-positive results. We have confirmed that these two mAbs could be applied to iELISA, IFA and western blot assays to detect SRLVs p28 or Gag precursor proteins. We also confirmed that the epitope sequences recognized by these two mAbs are relatively conserved among different SRLVs subtypes. These results suggest that these two monoclonal antibodies can be used as important supporting tools for basic research and detection of SRLVs. Previously, Ma et al. prepared mAbs against the HIV-1 CA protein, p24, which have been successfully used in colloidal gold immunochromatographic assays (Ma et al., 2016). Moreover, our laboratory has successfully established an antigen capture enzyme-linked immunosorbent assay (AC-ELISA) based on two mAbs against the EIAV CA protein, p26, for the quantification of EIAV (Hu et al., 2014).

In the present study, we found that the two identified mAbs recognize both prokaryotically and eukaryotically expressed p28 proteins, suggesting that they bind to linear epitopes. To determine the p28 linear B-cell epitope recognized by the two mAbs, a series of GST-fused p28 segments expressed in *E. coli* were generated. Identification with WB showed that the G8F7 antibody specifically recognized the ^61^GNRAQKELIQGKLNEEA^77^ epitope, and that the A10C12 antibody was specific for the ^187^CQKQMDRVLGTRVQQATVEEKMQACRDVCR^212^ epitope. Previous studies have shown that SRLVs p28 contains two immunodominant epitopes (designated as epitopes 2 and 3) (Rosati et al., 1999; L'homme et al., 2015). Interestingly, the epitopes identified in this study, E61-77 and E187-212, overlap those two immunodominant epitopes (L'homme et al., 2015). E61-77 completely overlaps the N-terminus of epitope 2, and the C-terminus of E187-212 overlaps the N-terminus of epitope 3 by 14 amino acids (aa 199–212) (Supplementary Figure S1). Unfortunately, the two epitopes identified in this study do not react with the few SRLVs-positive sera in our laboratory that have been shown to react with full-length p28 using WB (results not shown). E61-77 is highly conserved among various isolates of SRLVs, and contains only one dominant mutation site (K72R), but this mutation does not interfere with recognition by G8F7. While there is variability observed in the E187-212 among SRLVs isolates, the majority of mutant epitopes were recognized by A10C12. One mutant epitope, from an SRLVs strain isolated from *Ovis aries* in Jordan, was not recognized. These findings suggest that the G8F7 and A10C12 may possess broad-spectrum reactivity against the circulating strains of SRLVs, and that they have potential for the development of SRLVs assays.

In summary, we developed two mAbs against SRLVs p28 and characterized the epitope sequences they recognized. These two mAbs were successfully used in IFA, WB, and ELISA for the detection of p28. Our findings contribute to basic research on SRLVs and to the development of diagnostic methods for the detection of SRLVs.

## Data availability statement

The datasets presented in this study can be found in online repositories. The names of the repository/repositories and accession number(s) can be found in the article/Supplementary material.

## Ethics statement

The animal study was approved by Animal Care and Ethics Committees of Harbin Veterinary Research Institute, Chinese Academy of Agricultural Sciences. The study was conducted in accordance with the local legislation and institutional requirements.

## Author contributions

XM: Conceptualization, Data curation, Formal analysis, Investigation, Methodology, Writing – original draft. MG: Conceptualization, Data curation, Formal analysis, Investigation, Methodology, Project administration, Writing – original draft. XZ: Investigation, Writing – original draft. WM: Investigation, Visualization, Writing – original draft. FX: Resources, Writing – original draft. X-FW: Conceptualization, Formal analysis, Project administration, Writing – original draft, Writing – review & editing. XW: Conceptualization, Funding acquisition, Project administration, Validation, Writing – original draft, Writing – review & editing.

## Footnotes

^1^ http://weblogo.berkeley.edu/logo.cgi
