# Supplementary material for: Identification and characterization of linear epitopes of monoclonal antibodies against the capsid proteins of small ruminant lentiviruses
Source: Front Microbiol. 2024 Aug 1;15:1452063. doi: 10.3389/fmicb.2024.1452063 (PMC11325181; doi:10.3389/fmicb.2024.1452063)
Supplement: Supplementary file 1 [file Presentation_1.pdf]

Supplementary Material

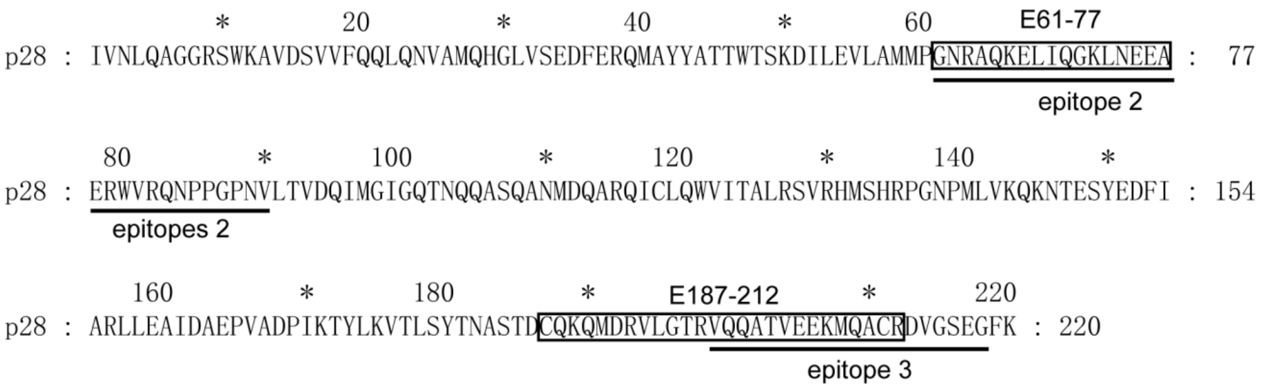

Supplementary Figure 1. SRLVs p28 amino acid sequence (GenBank accession number: PP854830). The previously described immunological epitopes (epitope 2 and epitope 3) are underlined, the linear epitopes (E61-77 and E187-212) identified in this study are boxed.
